# Comorbidity study of borderline personality disorder: applying association rule mining to the Taiwan national health insurance research database

**DOI:** 10.1186/s12911-016-0405-1

**Published:** 2017-01-11

**Authors:** Cheng-Che Shen, Li-Yu Hu, Ya-Han Hu

**Affiliations:** 1 Department of Psychiatry, Kaohsiung Veterans General Hospital, Kaohsiung, Taiwan; 2 Department of Psychiatry, Chiayi Branch, Taichung Veterans General Hospital, Chiayi, Taiwan; 3 Department of Information Management and Institute of Healthcare Information Management, National Chung Cheng University, Chiayi, Taiwan; 4 School of Medicine, National Yang-Ming University, Taipei, Taiwan

**Keywords:** Borderline personality disorder, Comorbidity, National health insurance database, Data mining association rules

## Abstract

**Background:**

Borderline personality disorder (BPD) is a complex clinical state with highly polymorphic symptoms and signs. Studies have demonstrated that people with a BPD diagnosis are likely to have numerous co-occurring psychiatric disorders and physical comorbidities. The aim of our study was to obtain further insight about the associations among comorbidities of BPD and to demonstrate the practicality of using association rule mining (ARM) technique in clinical databases.

**Methods:**

A retrospective case–control study was conducted on information of 1460 patients (292 BPD patients and 1168 control patients) selected from the Taiwan National Health Insurance Research Database. Information on physical and psychiatric comorbidities, which were diagnosed within 3 years before and after enrollment, was collected. A logistic regression model was used to calculate the odds ratios of comorbidities between patients with and without BPD. ARM technique was used to study the associations of BPD and two or more psychiatric comorbidities.

**Results:**

We classified physical comorbidities into 13 categories according to the International Classification of Diseases, Ninth Revision, Clinical Modification system, and the results indicated that the 12 categories were more common in the BPD patients than in the control patients (except congenital anomalies). However, psychiatric comorbidities, including depressive disorder, bipolar disorder, anxiety disorder, sleep disorder, substance use disorder, and mental retardation were more common in the BPD patients than in the control patients. Furthermore, the associations of BPD and two or more comorbidities were evaluated.

**Conclusion:**

Most physical and psychiatric disorders were more common in the BPD patients than in the control patients. Because the failure to remit from BPD is associated with suffering from chronic physical conditions and because psychiatric comorbidities may lead to delays in diagnosis of BPD, clinicians caring for people with BPD should be aware of possible comorbidities.

## Background

Borderline personality disorder (BPD) is one of the most prevalent mental disorders, and it accounts for approximately 2.7–5.9% of the world’s general population [1, 2], with slightly higher rates among women, people younger than 30 years of age, and people who are separated or divorced [3]. This disorder constitutes 10% of mental health clients observed in outpatient clinical settings and 15–20% of those in inpatient settings [4, 5]. BPD is a severe and complex psychological disorder characterized by pervasive instability in regulating emotions, self-image, interpersonal relationships, and impulse control [6]. BPD is associated with high psychosocial and socioeconomic costs [7]. The economic burden of diseases associated with BPD is higher than that of those associated with depression and comparable to that of patients with schizophrenia [8]. BPD is associated with severe functional impairment, substantial treatment use, and high rates of mortality by suicide [9–11]. For example, a study reported that among people with high rates of inpatient psychiatric hospitalization, 19% met the criteria for BPD based on clinical record diagnosis [12]. In addition, patients with BPD are frequently encountered in emergency departments, where they present following a suicide attempt or threatened suicide. More than 500 000 such events occur each year in the United States [13], and 10% of BPD patients die by suicide [14].

BPD is a complex clinical state with highly polymorphic symptoms and signs. Studies have demonstrated that people with a BPD diagnosis are likely to have numerous co-occurring psychiatric disorders, such as mood disorder, anxiety disorder, substance use disorder, and other personality disorders [3, 15–19]. However, most of these comorbidity studies of BPD have been conducted according to the prevalence of the co-occurrence of two diagnoses, and an entire association among three or more comorbid diseases remains under investigation. The association of comorbidities of a disorder is important but there are only some studies focusing on this issue [20, 21]. In addition, little empirical evidence has been found to demonstrate the association between BPD and physical comorbidities.

Association rule mining (ARM) has been used in many studies with clinical dataset [21–31]. In 2003, Chen [31] first introduced an application of ARM to Taiwan National Health Insurance Research Database (NHIRD) and showed the co-prescription patterns for antacids among enrollees. In 2009, Chiu [21] applied ARM to study the association among two or more comorbid disease of attention deficit hyperactivity disorder (ADHD). Therefore, we believe that it has potential practicality of using ARM technique in NHIRD and ARM could be applied in studying the comorbidities of BPD.

The aim of the present analysis was to explore the physical and psychiatric comorbidities of BPD by using the NHIRD and the ARM technique. The odds ratios (ORs) of comorbidities between patients with and without BPD were also surveyed. Our objective was to obtain further insight about the associations among comorbidities of BPD.

## Methods

### Data source

Taiwan instituted the National Health Insurance (NHI) program, a mandatory single-payer program that offers comprehensive medical care coverage, including outpatient, inpatient, emergency, and traditional Chinese medicine services, to almost 98% of residents on March 1, 1995 [32]. Moreover, as of 2014, 99.9% of Taiwan’s population was enrolled and foreigners in Taiwan were also eligible for the NHI program.

Since 1996, the NHI reimbursement data in Taiwan have been transferred to the National Health Research Institute (NHRI) for further management and organization. In addition, as part of these efforts, the work of NHRI has resulted in establishing a national health care database called NHIRD, which contains comprehensive information on clinical practices, including patients’ demographic characteristics, medical expenditure, prescription claims data, surgery code, treatment code, and diagnostic codes based on the International Classification of Disease, Ninth Revision, Clinical Modification (ICD-9-CM).

The use of NHIRD is limited to research purposes only. Researchers must follow the Computer-Processed Personal Data Protection Law (http://www.winklerpartners.com/?p=987) and the regulations of NHRI. In addition, an agreement must be signed by the researchers and their supervisor upon application submission. All applications for the databases release would be reviewed for approval by experts in NHIR. Furthermore, confidentiality is also maintained based on the directives of the Bureau of NHI. In the current study, the Longitudinal Health Insurance Database 2005 (LHID 2005), which is a dataset released by the NHRI, was used as the data source. The LHID 2005 contains all the original claims data of 1,000,000 beneficiaries enrolled in year 2005 randomly sampled from the year 2005 Registry for Beneficiaries of the NHIRD, where registration data of everyone who was a beneficiary of the NHI program during the period of January 1st 2005 to January 1st, 2006. There are approximately 25.68 million individuals in this registry. All the registration and claims data of these 1,000,000 individuals collected by the NHI program constitute the LHID 2005. The NHRI reported that no significant difference exists in the average insured payroll-related amount, sex distribution, or age distribution between patients in the LHID 2005 and those in the NHIRD.

### Study population

The data extracted from the LHID 2005 were used to conduct a retrospective case–control study on patients who were newly diagnosed with BPD (ICD-9-CM code: 301.83) by a psychiatrist between January 1, 2003 and December 31, 2006. For each BPD patient, 4 age- and sex-matched control patients without BPD were randomly selected from the LHID 2005 between 2003 and 2006. The random assignment procedures were performed by SAS statistical software and were based on the random numbers which were generated from the uniform distribution. Information on physical and psychiatric comorbidities, which were diagnosed within 3 years before and after enrollment, was collected. In this study, all comorbidities were categorized according to the original classification of the ICD-9-CM system. Details regarding psychiatric disorders including depressive disorder, bipolar disorder, anxiety disorder, substance use disorder (e.g., alcohol use disorder, opioid use disorder, and amphetamine use disorder), sleep disorder, eating disorder, autistic spectrum disorder, mental retardation, and ADHD were categorized for the ARM analysis.

### Statistical analysis

The prevalence rate of comorbidities in the BPD and control patients was calculated, and independent *t* and chi-squared tests were used to examine the differences in the demographic characteristics between the BPD and control patients. A univariable logistic regression model was also used to calculate the ORs of physical and psychiatric comorbidities between the BPD and control patients. In addition, although the coverage rate of the NHI system in Taiwan is up to 99%, there is still a very low incidence of missing data in the dataset. However, in our study, the missingness was unrelated to the variables, as so called missing completely at random. Therefore, we have adopted the most commonly complete case analyses method to accomondate these missing data-to simply exclude those participants in our dataset who have any data missing. SAS statistical software for Windows, Version 9.3 (SAS Institute, Cary, NC, USA), was used for data extraction, computation, data linkage, processing, and sampling. All other statistical analyses were conducted using SPSS statistical software for Windows, Version 20 (IBM, Armonk, NY, USA). Comparisons with *P* < .05 indicated statistically significant relationships.

### Association rule mining

ARM is one of the most useful methods for discovering patterns or extracting co-occurrences from transactional databases. Recently, ARM has been applied in clinical data analysis [21, 33]. A collection of entire diagnoses can be defined as a set of items, and enrollees’ clinical records can be represented as transactions, which include their historical combination sets. Therefore, the basic concept of ARM used in clinical data analysis can be outlined as follows.

Let *I* be a complete set of diagnoses (i.e., *items* in conventional ARM) and *T* = {*T*_1_, *T*_2_,…, *T*_*m*_} be a set of enrollees’ clinical records (i.e., *transactions* in conventional ARM), where *T*_*i*_ (1 ≤ *i* ≤ *m*) is a set of diagnoses for enrollee *i* (ie, *T*_*i*_ ⊆ *I*). Given two nonoverlapping sets of diagnoses, *X* and *Y* (*X* ⊂ *I*, *Y* ⊂ *I* and *X*∩*Y* = ϕ), an association rule is an implication of the form *X* → *Y* (which is read as “*X* implies *Y*”), indicating that if a set of diagnoses *X* occurs, then a set of diagnoses *Y* also occurs in the enrollee’s clinical record [34]. *X* and *Y* represent the antecedent and consequent of the rule, respectively.

Two measures, *support* and *confidence*, must be assessed in the mining process to discover association rules. The rule *X* → *Y* has *support s* in *T* if *s*% of the enrollees’ clinical records in *T* contains *X*∪*Y*; the rule has confidence *c* if *c*% of enrollees’ clinical records in *T* that support *X* also support *Y*. The confidence *c* could be also expressed as Probability (*Y*|*X*) [P (*Y*|*X*)]. Given a user-specified minimum support (called *minsup*) and minimum confidence (called *minconf*), the goal of ARM is to discover all association rules that have support and confidence greater than *minsup* and *minconf*, respectively.

Although the support-confidence framework for ARM has been widely studied in the literature [21, 33], it is a challenging task to set *minsup* and *minconf* simultaneously in real world application [35, 36]. To address this issue, we disregard *minsup* and *minconf* and consider only the *interestingness* of rules in ARM. Previous studies indicate that using interestingness measures can quickly evaluate the quality of rules and thus facilitate the rule consolidation process [37]. This study chooses the *lift* as the interestingness measure. The lift of the rule *X* → *Y* is defined as follows:

$$ lift\left(X\to Y\right)=\frac{c\left(X\to Y\right)}{s(Y)}=\frac{P\left(Y\left|X\right.\right)}{P(Y)}=\frac{P\left(X,Y\right)}{P(X)\times P(Y)} $$

The value of lift means that how much does the joint probability *P* (*X*, *Y*) deviate from the independence assumption *P* (*X*) × *P* (*Y*). Based on the above equation, we can interpret the measure as follows:

$$ lift\left(X\to Y\right)\left\{\begin{array}{c}\hfill >1,\hfill \\ {}\hfill =1,\hfill \\ {}\hfill <1,\hfill \end{array}\right.\begin{array}{c}\hfill \hfill \\ {}\hfill \hfill \\ {}\hfill \hfill \end{array}\begin{array}{c}\hfill \mathrm{if}\ X\ \mathrm{and}\ Y\ \mathrm{are}\ \mathrm{positively}\ \mathrm{correlated}\hfill \\ {}\hfill \mathrm{if}\ X\ \mathrm{and}\ Y\ \mathrm{are}\ \mathrm{independent}\kern3.25em \hfill \\ {}\hfill \mathrm{if}\ X\ \mathrm{and}\ Y\ \mathrm{are}\ \mathrm{negatively}\ \mathrm{correlated}\hfill \end{array} $$

The objective of this study was to determine the main psychiatric comorbidity of BPD by using ARM. In this study, analyses of ARM were conducted using Weka 3.6 open-source machine learning software (www.cs.waikato.ac.nz/ml/weka). The Apriori module in WEKA was used to discover interesting association rules relating to comorbidity of BPD. To apply the lift metric in Apriori module, we set both *minsup* and *minconf* as 0% and specify the metric type as lift. The minimum value of lift is defined as 1 in order to discover the rules having positive correlation between their antecedent and consequent.

Based on the above setting, all the interesting association rules (ie, *lift* > 1) will be outputted in descending order by their lift values in WEKA. Although the lift metric can prune less meaningful rules during the mining process, the number of generated rules is still huge because of disregarding *minsup* and *minconf* thresholds. In this study, we confine the number of generate rules in WEKA. Specifically, we generate top-*k* (*k* = 10,000) interesting rules by specifying parameter *k* in WEKA.

In order to evaluate whether the discovered association rules hold in general, we partitioned the collected data into training and testing sets. Specifically, two third of patients were randomly selected as the training set to discover association rules and the remaining one third of patients (testing set) were used to validate the discovered association rules [38, 39].

Because small supports were noted in some discovered association rules, bootstrap simulation was also used to validate the discovered association rules. Of the 292 BPD patients included in our study, a random sample of 292 BPD patients is drawn with replacement (Therefore, this sample will include some BPD patients multiple times, and other BPD patients will be excluded at random.). The 292 BPD patients were matched to 4 controls according to age and sex, resulting in a bootstrap sample. The association rules on this bootstrap sample were evaluated. Above steps were repeated 1000 times to create 1000 bootstrap samples. The mean support, confidence, lift, and ORs with 95% confidence interval (CI) of 1000 bootstrap samples were calculated.

## Results

### Patient selection

Our study included 292 BPD patients and 1168 control patients, 65% of whom were women. The median age at enrollment was 25 years (interquartile range, 21–33 y). Table 1 shows comparisons of demographic variables between the BPD and control patients.

**Table 1:** Characteristics of patients with borderline personality disorder (BPD) and comparison subjects

	BPD, *N* = 292 (%)	Control, *N* = 1168 (%)	*P* values
Sex			0.999
Male	102 (34.9)	408 (34.9)	
Female	190 (65.1)	760 (65.1)	
Age (years)^a^	25 (21–33)	25 (21–33)	0.999
Distribution of age			0.999
0–19	37 (12.7)	148 (12.7)	
20–39	226 (77.4)	904 (77.4)	
40–59	29 (9.9)	116 (9.9)	

^a^Median (interquartile range)

### Odds ratios of physical comorbidities between the borderline personality disorder and control patients

The results of the univariable logistic regression analysis (Table 2) indicated that the ORs for having 12 categories of physical comorbidities, except congenital anomalies, were greater for the BPD patients than for the control patients, particularly for comorbidities resulting from injury and poisoning (OR = 4.84, 95% CI = 3.45–6.78). In addition, the ORs for having comorbidities of the respiratory system (OR = 4.09, 95% CI = 1.49–11.2); digestive system (OR = 3.32, 95% CI = 1.33–8.31); genitourinary system (OR = 14.7, 95% CI = 8.15–26.5); endocrine, metabolic, and immune system (OR = 2.68, 95% CI = 1.43–5.01); neoplasm (OR = 2.58, 95% CI = 1.33–5.01); and blood and blood-forming system (OR = 4.08, 95% CI = 1.54–10.8) were greater for female BPD patients than for male BPD patients.

**Table 2:** Physical comorbidity rates of borderline personality disorder (BPD) cases and non-BPD controls with diseases categorized in ICD-9-CM coding system

		BPD				BPD (total)^c^ (%)	Non-BPD (total)^d^ (%)	OR	95% CI
Category of comorbid disease	ICD 9 CM codes	Female (%)	Male (%)	OR^a^	95% CI^b^				
Respiratory system	460–519	96.8	88.2	4.09	1.49–11.2^e^	93.8	88.2	2.04	1.23–3.39^e^
Nervous system and sense organs	320–389	72.6	64.7	1.45	0.86–2.43	69.9	52.9	2.15	1.63–2.83^e^
Digestive system	520–579	95.8	87.3	3.32	1.33–8.31^e^	92.8	81.9	2.85	1.78–4.55^e^
Skin and subcutaneous tissue	680–709	72.6	65.7	1.39	0.83–2.33	70.2	56.0	1.85	1.41–2.44^e^
Injury and poisoning	800–999	84.7	84.3	1.03	0.53–2.01	84.6	53.2	4.84	3.45–6.78^e^
Infectious and parasitic	001–139	52.1	42.2	1.49	0.92–2.43	48.6	35.6	1.71	1.32–2.22^e^
Genitourinary system	580–629	81.1	22.5	14.7	8.15–26.5^e^	60.6	46.3	1.78	1.37–2.31^e^
Musculoskeletal system	710–739	65.3	61.8	1.16	0.71–1.92	64.0	42.1	2.45	1.88–3.19^e^
Congenital anomalies	740–759	3.68	2.94	1.26	0.32–4.99	3.42	1.63	2.14	0.99–4.66
Endocrine, metabolic and immunity	240–279	31.6	14.7	2.68	1.43–5.01^e^	25.7	14.3	2.07	1.52–2.82^e^
Neoplasm	140–239	27.4	12.7	2.58	1.33–5.01^e^	22.3	11.5	2.21	1.59–3.07^e^
Circulatory system	390–459	29.5	23.5	1.36	0.78–2.36	27.4	11.1	3.01	2.20–4.13^e^
Blood and blood-forming organs	280–289	17.4	4.90	4.08	1.54–10.8^e^	13.0	4.79	2.97	1.93–4.59^e^

^a^ *OR* odds ratio, ^b^ *CI* confidence interval, ^c^ Total BPD cases, ^d^ Total non-BPD controls, ^e^ Statistical significance

### Odds ratios of psychiatric comorbidities between the borderline personality disorder and control patients

The results of the univariable logistic regression analysis (Table 3) showed that the ORs for having depressive disorder (OR = 220, 95% CI = 133–364), bipolar disorder (OR = 261.1, 95% CI = 157–434), anxiety disorder (OR = 13.2, 95% CI = 9.53–18.4), substance use disorder (OR= 73.3, 95% CI = 24.2–253), sleep disorder (OR = 6.82, 95% CI = 5.00–9.29), and mental retardation (OR = 6.11, 95% CI = 2.43–15.3) were greater for BPD patients than for control patients. In addition, the ORs for having depressive disorder (OR = 2.16, 95% CI = 1.18–3.96), bipolar disorder (OR = 2.34, 95% CI = 1.21–4.53), anxiety disorder (OR = 1.87, 95% CI = 1.14–3.06), and sleep disorder (OR = 1.85, 95% CI = 1.11–3.09) were greater for female BPD patients than for male BPD patients, but the ORs for having substance use disorder (OR = 0.49.8, 95% CI = 0.27–0.92) and mental retardation (OR = 0.10, 95% CI = 0.01–0.89) were lower for female BPD patients than for male BPD patients.

**Table 3:** The comorbid psychiatry diseases of borderline personality disorder (BPD) cases and non-BPD controls

	BPD				BPD (total)^c^ (%)	Non-BPD (total)^d^ (%)	OR	95% CI
Comorbid psychiatry diseases	Female (%)	Male (%)	OR^a^	95% CI^b^				
Depressive disorder	86.3	74.5	2.16	1.18–3.96^e^	82.2	2.05	220	133–364^e^
Bipolar disorder	89.5	78.4	2.34	1.21–4.53^e^	85.6	2.23	261	157–434^e^
Anxiety disorder	52.6	37.3	1.87	1.14–3.06^e^	47.3	6.34	13.2	9.53–18.4^e^
Sleep disorder	44.7	30.4	1.85	1.11–3.09^e^	39.7	8.82	6.82	5.00–9.29^e^
Substance use disorder	13.2	23.5	0.49	0.27–0.92^e^	16.8	0.26	73.3	24.2–253^e^
Alcohol use disorder	8.42	20.6	0.36	0.18–0.72^e^	12.7	0.26	56.3	17.2–184.1^e^
Opioid use disorder	2.11	1.96	1.08	0.19–5.97	2.05	0	−	−
Amphetamine use disorder	3.16	0.98	3.29	0.39–27.7	2.40	0	−	−
ADHD	1.05	2.94	0.35	0.06–2.14	1.71	0	−	−
Mental retardation	0.53	4.9	0.10	0.01–0.89^e^	2.05	0.26	6.11	2.43–15.3^e^
Autistic spectrum disorder	0	0.53	−	−	0.34	0	−	−
Eating disorder	0	3.68	−	−	2.40	0	−	−

^a^ *OR* odds ratio, ^b^ *CI* confidence interval, ^c^Total BPD cases, ^d^Total non-BPD controls, ^e^Statistical significance

### Association rule mining for psychiatic comorbidities of borderline personality disorder

Table 4 lists the results of ARM for psychiatric comorbidities in training set. Thirteen association rules reached the selected thresholds. Those rules are as follows: BPD ⇒ bipolar disorder and anxiety disorder (support: 8.5%, confidence: 43%, lift: 4.41); BPD ⇒ depressive disorder and anxiety disorder (support: 8.2%, confidence: 41%, lift: 4.39); BPD ⇒ bipolar disorder and sleep disorder (support: 7.3%, confidence: 36%, lift: 4.37); BPD ⇒ depressive disorder and sleep disorder (support: 7.1%, confidence: 35%, lift: 4.41); BPD ⇒ anxiety disorder and sleep disorder (support: 4.9%, confidence: 25%, lift: 3.37); BPD ⇒ bipolar disorder, anxiety disorder, and sleep disorder (support: 4.6%, confidence: 23%, lift: 4.49); BPD ⇒ depressive disorder, anxiety disorder, and sleep disorder (support: 4.4%, confidence: 22%, lift: 4.47); BPD ⇒ bipolar disorder and substance use disorder (support: 3.2%, confidence: 16%, lift: 4.99); BPD ⇒ depressive disorder and substance use disorder (support: 3.0%, confidence: 15%, lift: 4.99); BPD ⇒ depressive disorder, anxiety disorder, and substance use disorder (support: 1.6%, confidence: 8.2%, lift: 4.99); BPD ⇒ substance use disorder and sleep disorder (support: 1.5%, confidence: 7.7%, lift: 4.99); BPD ⇒ depressive disorder, substance use disorder, and sleep disorder (support: 1.4%, confidence: 7.4%, lift: 4.99); and BPD ⇒ bipolar disorder, substance use disorder, and sleep disorder (support: 1.4%, confidence: 7.2%, lift: 4.99).

**Table 4:** Results of association rule mining of comorbid psychiatric disorders among Borderline personality disorder (BPD) cases in training set

Relations	Support (%)	Confidence (%)	Lift	Rule	BPD^a^	Non-BPD^b^	OR^c^	95% CI^d^
3	8.5	43	4.41	BPD ➔ Bipolar disorder & Anxiety disorder	84	11	52.8	27.3–102^e^
3	8.2	41	4.39	BPD ➔ Depressive disorder & Anxiety disorder	80	11	48.5	25.1–93.9^e^
3	7.3	36	4.37	BPD ➔ Bipolar disorder & Sleep disorder	70	10	43.0	21.6–85.7^e^
3	7.1	35	4.41	BPD ➔ Depressive disorder & Sleep disorder	68	9	45.7	22.3–94.0^e^
3	4.9	25	3.37	BPD ➔ Anxiety disorder & Sleep disorder	49	23	11.0	6.51–18.6^e^
4	4.6	23	4.49	BPD ➔ Bipolar disorder & Anxiety disorder & Sleep disorder	45	5	46.4	18.1–119^e^
4	4.4	22	4.47	BPD ➔ Depressive disorder & Anxiety disorder & Sleep disorder	43	5	43.7	17.0–112^e^
3	3.2	16	4.99	BPD ➔ Bipolar disorder & Substance use disorder	31	0	−	−
3	3.0	15	4.99	BPD ➔ Depressive disorder & Substance use disorder	29	0	−	−
4	1.6	8.2	4.99	BPD ➔ Depressive disorder & Anxiety disorder & Substance use disorder	16	0	−	−
3	1.5	7.7	4.99	BPD ➔ Substance use disorder & Sleep disorder	15	0	−	−
4	1.4	7.2	4.99	BPD ➔ Depressive disorder & Substance use disorder & Sleep disorder	14	0	−	−
4	1.4	7.2	4.99	BPD ➔ Bipolar disorder & Substance use disorder & Sleep disorder	14	0	−	−

^a^Number of BPD patients having the comorbidities in the rule

^b^Number of non-BPD patients having the comorbidities in the rule

^c^OR odds ratio; ^d^CI confidence interval; ^e^Statistical significance

Table 5 shows results of applying rules discovered in training set to testing set Those results are as follows: BPD ⇒ bipolar disorder and anxiety disorder (support: 9.1%, confidence: 45%, lift: 4.80); BPD ⇒ depressive disorder and anxiety disorder (support: 8.7%, confidence: 43%, lift: 4.90); BPD ⇒ bipolar disorder and sleep disorder (support: 7.2%, confidence: 36%, lift: 4.75); BPD ⇒ depressive disorder and sleep disorder (support: 7.0%, confidence: 35%, lift: 4.88); BPD ⇒ anxiety disorder and sleep disorder (support: 5.4%, confidence: 27%, lift: 3.96); BPD ⇒ bipolar disorder, anxiety disorder, and sleep disorder (support: 4.9%, confidence: 25%, lift: 4.82); BPD ⇒ depressive disorder, anxiety disorder, and sleep disorder (support: 4.7%, confidence: 24%, lift: 5.02); BPD ⇒ bipolar disorder and substance use disorder (support: 2.7%, confidence: 13%, lift: 5.02); BPD ⇒ depressive disorder and substance use disorder (support: 2.7%, confidence: 13%, lift: 5.02); BPD ⇒ depressive disorder, anxiety disorder, and substance use disorder (support: 1.0%, confidence: 5.2%, lift: 5.02); BPD ⇒ substance use disorder and sleep disorder (support: 2.1%, confidence: 10%, lift: 5.02); BPD ⇒ depressive disorder, substance use disorder, and sleep disorder (support: 2.1%, confidence: 10%, lift: 5.02); and BPD ⇒ bipolar disorder, substance use disorder, and sleep disorder (support: 2.1%, confidence: 10%, lift: 5.02). The results of association rules including support, confidence, and lift were similar in training set and testing set.

**Table 5:** Results of applying rules discovered in training set to testing set

Relations	Support (%)	Confidence (%)	Lift	Rule	BPD^a^	Non-BPD^b^	OR^c^	95% CI^d^
3	9.1	45	4.80	BPD ➔ Bipolar disorder & Anxiety disorder	44	2	161	37.9–684^e^
3	8.7	43	4.90	BPD ➔ Depressive disorder & Anxiety disorder	42	1	297	40.1–2202^e^
3	7.2	36	4.75	BPD ➔ Bipolar disorder & Sleep disorder	35	2	109.5	25.7–467^e^
3	7.0	35	4.88	BPD ➔ Depressive disorder & Sleep disorder	34	7	29.5	12.5–69.5^e^
3	5.4	27	3.96	BPD ➔ Anxiety disorder & Sleep disorder	26	7	20.0	8.38–47.9^e^
4	4.9	25	4.82	BPD ➔ Bipolar disorder & Anxiety disorder & Sleep disorder	24	1	128	17.0–960^e^
4	4.7	24	5.02	BPD ➔ Depressive disorder & Anxiety disorder & Sleep disorder	23	0	−	−
3	2.7	13	5.02	BPD ➔ Bipolar disorder & Substance use disorder	13	0	−	−
3	2.7	13	5.02	BPD ➔ Depressive disorder & Substance use disorder	13	0	−	−
4	1.0	5.2	5.02	BPD ➔ Depressive disorder & Anxiety disorder & Substance use disorder	5	0	−	−
3	2.1	10	5.02	BPD ➔ Substance use disorder & Sleep disorder	10	0	−	−
4	2.1	10	5.02	BPD ➔ Depressive disorder & Substance use disorder & Sleep disorder	10	0	−	−
4	2.1	10	5.02	BPD ➔ Bipolar disorder & Substance use disorder & Sleep disorder	10	0	−	−

^a^Number of BPD patients having the comorbidities in the rule

^b^Number of non-BPD patients having the comorbidities in the rule

^c^OR odds ratio; ^d^CI confidence interval; ^e^Statistical significance

Furthermore, the results of bootstrap simulation were presented in Table 6 and the results revealed that the association rules discovered in our study were statistically significant.

**Table 6:** Mean support, confidence, lift, and odds ratio (ORs) of association rules of comorbid psychiatric disorders among Borderline personality disorder (BPD) cases of 1000 bootstrap samples

Relations	Support (%)	95% CI^a^	Confidence (%)	95% CI^a^	Lift	95% CI^a^	Rule	OR	95% CI^a^
3	8.69	8.65–8.73^b^	43.4	43.2–43.7^b^	4.39	4.38–4.40^b^	BPD ➔ Bipolar disorder & Anxiety disorder	53.0	52.1–53.9^b^
3	8.35	8.31–8.38^b^	41.7	41.6–41.9^b^	4.41	4.40–4.42^b^	BPD ➔ Depressive disorder & Anxiety disorder	53.7	52.8–54.62^b^
3	7.26	7.22–7.29^b^	36.3	36.1–36.5^b^	4.33	4.32–4.34^b^	BPD ➔ Bipolar disorder & Sleep disorder	42.8	42.0–43.6^b^
3	7.04	7.01–7.08^b^	35.2	35.1–35.1^b^	4.35	4.34–4.36^b^	BPD ➔ Depressive disorder & Sleep disorder	43.8	43.0–44.6^b^
3	5.07	5.04–5.10^b^	25.3	25.2–25.5^b^	3.56	3.55–3.58^b^	BPD ➔ Anxiety disorder & Sleep disorder	13.4	13.2–13.6^b^
4	4.73	4.69–4.76^b^	23.6	23.5–23.8^b^	4.40	4.39–4.41^b^	BPD ➔ Bipolar disorder & Anxiety disorder & Sleep disorder	42.4	41.2–43.6^b^
4	4.52	4.49–4.55^b^	22.6	22.4–22.7^b^	4.39	4.38–4.40^b^	BPD ➔ Depressive disorder & Anxiety disorder & Sleep disorder	41.8	40.7–43.0^b^
3	3.01	2.99–3.04^b^	15.1	14.9–15.2^b^	4.91	4.90–4.91^b^	BPD ➔ Bipolar disorder & Substance use disorder	−	−
3	2.88	2.85–2.90^b^	14.4	14.3–14.5^b^	4.90	4.90–4.91^b^	BPD ➔ Depressive disorder & Substance use disorder	−	−
4	14.3	14.1–14.5^b^	7.15	7.06–7.25^b^	4.85	4.84–4.86^b^	BPD ➔ Depressive disorder & Anxiety disorder & Substance use disorder	−	−
3	1.71	1.69–1.73^b^	8.55	8.45–8.65^b^	4.80	4.79–4.81^b^	BPD ➔ Substance use disorder & Sleep disorder	−	−
4	1.64	1.62–1.66^b^	8.21	8.11–8.32^b^	4.87	4.86–4.88^b^	BPD ➔ Depressive disorder & Substance use disorder & Sleep disorder	−	−
4	1.64	1.62–1.66^b^	8.21	8.11–8.32^b^	4.87	4.86–4.88^b^	BPD ➔ Bipolar disorder & Substance use disorder & Sleep disorder	−	−

^a^CI confidence interval; ^b^Statistical significance

## Discussion

The key findings in our study are outlined as follows: (1) The 12 categories of physical comorbidities classified according to the ICD-9-CM system were more common in the BPD patients than in the control patients (except congenital anomalies); (2) the psychiatric comorbidities including depressive disorder, bipolar disorder, anxiety disorder, sleep disorder, substance use disorder, and mental retardation were more common in the BPD patients than in the control patients; (3) depressive disorder, bipolar disorder, anxiety disorder, and sleep disorder were more prevalent for female BPD patients than for male BPD patients, but substance use disorder and mental retardation were less prevalent for female BPD patients than for male BPD patients; (4) the associations of BPD and two or more comorbidities were demonstrated in our work by using ARM.

In this study, we observed that the 12 categories of physical comorbidities classified according to the ICD-9-CM system were more common in the BPD patients than in the control patients. The results were consistent with those of previous studies. A national study in the United States showed that BPD was significantly associated with arteriosclerosis or hypertension, hepatic disease, cardiovascular disease, gastrointestinal disease, arthritis, venereal disease, and “any assessed medical condition” [40]. In addition, McWilliams et al. demonstrated that BPD symptoms were positively associated with chronic spinal pain, frequent headaches, and other chronic pain conditions [41]. Because the failure to remit from BPD seemed to be associated with a heightened risk of suffering from chronic physical conditions [42], clinicians caring for people with personality disorders must be aware of possible medical comorbidities associated with such disorders [43]. Furthermore, gender differences in physical comorbidity profiles were noted in our study. Comorbidities of the respiratory system, digestive system, genitourinary system, endocrine, metabolic, and immune system, neoplasm, and blood and blood-forming system were more prevalent for female BPD patients than for male BPD patients. Because BPD is more common in women than in men [3], more attention should be focused on the evaluation of physical comorbidities in patients with BPD.

We observed that the risk of depressive disorder, bipolar disorder, anxiety disorder, substance use disorder, and sleep disorder in BPD patients was higher than that in non-BPD patients; this finding is consistent with those of previous studies [3, 16–18]. BPD is commonly associated with high rates of psychiatric comorbidity. In urban primary care patients, the majority (91%) of patients screening positive for BPD satisfied the criteria for at least one current Diagnostic and Statistical Manual of Mental Disorders-IV Axis I diagnosis [19]. Zimmerman et al. reported that people with BPD satisfy the criteria for an average of 3.0–3.4 current Axis I disorders and 4.2–4.8 lifetime Axis I disorders [16]. In addition, they revealed that compared with non-BPD patients, BPD patients more frequently receive diagnoses of mood, anxiety, substance use, and somatoform disorders [16], which is highly compatible with the results of our study. Because psychiatric comorbidities are associated with a heightened risk of suicidal and nonsuicidal self-injury among people with BPD [44–46] and have been found to reduce the likelihood of achieving remission from BPD [47], determining potential psychiatric comorbidities in BPD patients is crucial.

The diagnosis of BPD in patients with mental retardation, where developmental brain abnormality is inherent, has rarely been reported in the literature [48, 49]. In this study, we found that the risk of mental retardation in the BPD patients was higher than that in the control patients. To our knowledge, this is the first report regarding this association, and our finding requires further confirmation.

In the field of psychiatry, several articles have discussed the comorbidity rates of BPD with “only one” other disease. However, few have discussed the comorbidity rates of three or more diseases. We used ARM in our study for two main reasons. First, ARM enabled us to observe the associations among three or more diagnoses simultaneously. Second, the value of confidence is arithmetically synonymous with the “comorbidity rate” in epidemiology. Using ARM, we determined that BPD was highly concurrent with “bipolar disorder and anxiety disorder” (43.49%), “depressive disorder and anxiety disorder” (41.49%), “bipolar disorder and sleep disorder” (36.30%), “depressive disorder and sleep disorder” (35.27%), “anxiety disorder and sleep disorder” (25.34%), “bipolar disorder, anxiety disorder, and sleep disorder” (confidence 23.63%), “depressive disorder, anxiety disorder, and sleep disorder” (22.60%), “bipolar disorder and substance use disorder” (15.07%), “depressive disorder and substance use disorder” (14.38%), “depressive disorder, anxiety disorder, and substance use disorder” (8.2%), “substance use disorder and sleep disorder” (7.7%), “depressive disorder, substance use disorder, and sleep disorder” (7.4%), and “bipolar disorder, substance use disorder, and sleep disorder” (7.2%). According to our results, most patients with BPD have two or more psychiatric comorbidities. Clinicians caring for people with BPD must be aware of this and determine potential psychiatric comorbidities in detail.

The strength of our study is that the study design included an unbiased patient selection process. Because participation in the NHI is mandatory and because all residents of Taiwan can access health care with low copayments, referral bias is low in our study. Furthermore, we partitioned the collected data into training and testing sets and found that the discovered association rules were validated. However, our study has some limitations. First, information regarding the family history, lifestyle factors, and environmental factors of those with physical and psychiatric disorders are not included in the NHIRD, all of which may be associated with the prevalence of comorbidities. Second, in studies entailing the use of the NHIRD, how diagnostic classification has been conducted, particularly for psychiatric diagnoses, is unclear. Therefore, the diagnostic accuracy in our study could not be ascertained. Additional studies with patients diagnosed through structured interviews or standard diagnostic criteria should be conducted to investigate the association between comorbidities and BPD. Third, the duration of the observational period in our study might have been insufficient. In addition, different duration of observational period might be a confounding variable of our study. Future studies with longer and different observational periods are thus required. Fourth, the composition of our study subjects is different from the composition of general population and the support and lift of a rule in the case–control matched population may be different from the support and lift of the same rule in the entire population. The difference between our study population and another population of interest should thus be taken into account when applying our results in a different context. Finally, the categories of physical comorbidities in our study might be too broad and many minor diseases were enrolled which resulted in high disease prevalence. Further studies with finer disease categories are required to investigate physical comorbidities of BPD patients.

## Conclusions

Our population-based retrospective case–control study revealed that physical comorbidities were more common in the BPD patients than in the control patients. In addition, psychiatric comorbidities including depressive disorder, bipolar disorder, anxiety disorder, sleep disorder, substance use disorder, and mental retardation were more common in the BPD patients than in the control patients. Furthermore, the associations of BPD and two or more comorbidities were demonstrated using ARM. Population-based large prospective studies are required to further validate our findings.

## Abbreviations

ADHD: Attention deficit hyperactivity disorder
ARM: Association rule mining
BPD: Borderline personality disorder
CI: Confidence interval
ICD-9-CM: The international classification of disease, ninth revision, clinical modification
LHID: The longitudinal health insurance database
NHIRD: National health insurance research database
NHRI: National health research institutes
ORs: Odds ratios
